# Nurse Managers' Perceptions Related to Their Leadership Styles, Knowledge, and Skills in These Areas—A Viewpoint: Case of Health Centre Wards in Finland

**DOI:** 10.1155/2013/951456

**Published:** 2013-04-03

**Authors:** Soili Vesterinen, Marjo Suhonen, Arja Isola, Leena Paasivaara, Helena Laukkala

**Affiliations:** ^1^Lapland Hospital District, P.O. Box 8041, 96101 Rovaniemi, Finland; ^2^Department of Health Sciences, University of Oulu, P.O. Box 5000, 90014 University of Oulu, Finland

## Abstract

The purpose of this study was to explore nurse managers' perceptions related to their leadership styles, knowledge, and their skills in these areas in health centre wards in Finland. The data were collected from nurse managers (*n* = 252) in health centre hospitals in Finland using a structured questionnaire (response rate 63%). Six leadership styles—visionary, coaching, affiliate, democratic, commanding, and isolating—were reflected on. Almost all respondents in every age group considered four leadership styles—visionary, coaching, affiliate, and democratic—to be very important or important. Nurse managers estimated their knowledge and skills in leadership styles to be essentially fairly sufficient or sufficient. Nurse managers' abilities to reflect, understand, and, if necessary, change their leadership style influence the work unit's success and employees' job satisfaction. Nurse managers, especially new nurse managers, need more theoretic, evidence-based education to cope with these expectations and to develop their professional abilities. Together with universities, health care organizations should start planning nurse manager education programmes that focus on strategic issues, leadership, job satisfaction, challenging situations in leadership, change management, work unit management (e.g., economy, efficiency, and resources), and how the nurse managers consider their own wellbeing.

## 1. Introduction

In many European countries, the public health care system is facing challenges, such as problems in recruiting professionals [1, 2] and staff retention [3]. The situation in Finland is similar. One of the major challenges in Finland is how to secure adequate, trained personnel. The proportion of persons aged over 65 in the Finnish population is estimated to rise from 17 percent to 27 percent by 2040 [4]. Shortage of staff is an imminent threat: 22.5 percent of the employees in social and health care will reach the age of 67 by 2020 at the latest. Most of them will retire at that time, or even earlier [5]. At the same time, 26 percent of young Finnish nurses have often thought of leaving their profession [6].

Nurses' work in health centre wards is physically and mentally burdensome; consequently, nurse manager's leadership skills have a key role in influencing nurses' job satisfaction and their staying on in a work unit [7, 8]. An essential part of leadership skills is the use of different leadership styles [9–11]. Leadership styles can be seen as different combinations of tasks and transaction behaviours that influence people in achieving goals [12].

This paper focuses on nurse managers' leadership styles in health centre hospital wards in Finland, because the Finnish health care system is a strong institution, where health care services are offered to all citizens and funded by taxes [13]. Health care services in Finland are of high quality [14]. It is important to explore nurse managers' leadership styles especially in this context.

The public health care sector in Finland is currently under changes. In June 2010, the government approved a proposal to amend the current health care law with the aim of strengthening primary health care, promoting welfare and health, and enhancing availability and effective production of health services [15]. Public health care system in Finland consists of primary health care and specialized medical care. Primary care is offered in health centres, which often have a ward for inpatients.

Working in a health centre ward consists of shift work, evenings, nights, and weekends included. Most of the patients in health centre wards have many diseases and are old. In 2008, patients' average age was 75 years [16]. The importance of nurse managers' leadership skills is particularly emphasized in this work context. Essential nurse manager competencies for the future include among other things the ability to create an organization culture that combines high-quality health care and patient/employee safety and highly developed collaborative and team building skills [1]. One future challenge for nurse managers is to create a culture where nurses are not afraid of requiring an environment where they are recognized as invaluable partners in the organization [17]. Nurse managers' role is also essential for promoting evidence-based practice within organizations. Evidence-based practice is important for the professional practice of nursing for many reasons. Because nursing is a science and a profession, nursing practice should be grounded on the best available evidence, and the findings of nursing research should be translated into practice. Evidence-based nursing facilitates paying attention to efficiency (providing nursing care with the appropriate level of staffing) and effectiveness (achieving desired outcomes) in nursing [18]. All of these future challenges can be met with the help of using effective leadership styles [19]. Consequently, nurse managers' major challenges in Finnish health centre wards are related to leadership skills. A nurse manager with the readiness to observe his/her own behaviour and its effects on the work unit and employees can adjust to a better leadership style [12]. Goleman et al. [20] have described primal leadership, which consists of resonant and dissonant leadership styles, depending on the situation. It requires the leader to bring emotional intelligence (EI) to bear on his/her leadership. EI has been associated with positive empowerment processes as well as positive organizational results [21]. Resonant leadership styles have been identified as visionary, coaching, affiliate, and democratic styles, while dissonant leadership styles have been identified as pacesetting and commanding styles. Most leaders use both resonant and dissonant leadership styles [20].

In recent years, nurse manager's EI has been recognized as an important research area [22]. Nurse manager's EI leadership behaviour has a strong impact on nurses' empowerment and organizational commitment [23] as well as on team effectiveness and the quality of nursing care [24]. On the other hand, nurse managers with strong EI may not be able to empower their employees if their span of control is wide [25].

## 2. Materials and Methods

### 2.1. Aim and Objectives

This study is the fourth part of a larger investigation concerning nurse managers' leadership styles. The first part was a review of studies on nurse leaders' leadership styles in the years 1996–2006 [26]. In the second part the purpose of the study was to discover nurse managers' perceptions of their leadership styles and the factors influencing it [27]. The third part of the study consisted of exploring how nurses and superiors perceive nurse managers' leadership styles and the factors affected by leadership styles [28]. The aim of the present paper is to explore nurse managers' perceptions related to their leadership styles, knowledge, and their skills in these areas in health centre wards in Finland. The research questions are as follows.

- What is the prominent leadership style demonstrated by nurse managers as perceived by themselves in health centre wards?
- How important do nurse managers consider different leadership styles?
- How adequate do nurse managers consider their skills and knowledge in leadership styles? and how important do they consider these styles?
- What are the associations between background variables (age, education, length of work experience as nurse manager, and updating education) and nurse managers' perceptions of the importance of different leadership styles and their knowledge and skills in leadership styles?

### 2.2. Questionnaire

The questionnaire used in the study is based on the primal leadership model 20 and earlier research of Vesterinen et al. [27, 28]. Once the questionnaire had been drawn up, its content was evaluated by an expert panel (*n* = 6). The expert panel consisted of nurse managers working in different types of hospital wards. Based on the results, the questionnaire was modified by making two statements more explicit and by revising the language.

In the final questionnaire, the leadership styles of the nurse managers were charted in the following three fields. Background information was asked for by 12 statements. To examine participants' prominent leadership style, they were asked to indicate how significant and important they considered the named factors in their work as nurse managers (36 statements). The response alternatives followed a 5-point Likert scale (1 = not at all important, 2 = rather unimportant, 3 = rather important, 4 = important, and 5 = very important).

Furthermore, the participants were asked to estimate how adequate their knowledge and skills were in different fields of management (34 statements). The response alternatives followed a 4-point Likert scale (1 = not at all sufficient, 2 = not quite sufficient, 3 = rather sufficient, and 4 = sufficient). In this 34-statement instrument, there were 18 statements concerning leadership styles. In this paper, we report on the basis of these statements the results of the respondents' perceptions regarding their knowledge and skills in different leadership styles.

### 2.3. Data Collection

The target population comprised all nurse managers in health centre hospitals in Finland. First, the number of all nurse managers in different health centres registered in the database of the National institute for Health and Welfare in Finland was enquired (*n* = 1,317). After that, different health centres were divided into five groups based on the number of nurse managers using stratified sampling: very small (1-2 nurse managers), small (3-4 nurse managers), medium sized (5–9 nurse managers), large (10–29 nurse managers), and very large (30–85 nurse managers). For the nationwide study, health centre organizations were chosen from these five groups using sampling proportional to size. After that, the nurse managers were chosen using systematic sampling until the number of 400 nurse managers was reached [29]. As the organizations had very differing numbers of nurse managers, a minimum of one and a maximum of 63 respondents were included in the study. 

The data collection was conducted from November 2009 to April 2010. An application for a statement was submitted to the Local Research Ethics Committee. The administrations of the health centres were contacted via e-mail, and their willingness to participate in the study was enquired. Following approval from the administration of the health centre organizations (*n* = 108), the data for this study were collected using a mailed questionnaire. The respondents were reached via their work addresses or via a contact person. Questionnaires, information about the study, and return envelopes were mailed straight to the respondents or via the contact person who delivered the material further to the respondents. Participants were informed of the purpose of the study in the covering letter. They were told that their participation was voluntary and would be treated with confidentiality. The covering letter also included detailed contact information on how to reach the researcher. Returning the questionnaire to the researcher was organized by post, which ensured that the identity of the respondents remained unknown. The respondents had three weeks to respond to the questionnaire. The response rate of the study was 63%, which can be regarded as reasonable, considering that the questionnaire was sent by post. The nurse managers' background data are given in Table 1.

### 2.4. Data Analysis

The data were analysed using the SPSS 12.0 software (SPSS Inc., Chicago, IL, USA). Several variables were analysed using frequencies, mean scores, standard deviations, and descriptive statistics. 

Some of the background data variables (age, education, work experience in health care, work experience as nurse manager, and number of employees) were classified before the calculation of frequency and percentage distributions. Some of the statements concerning visionary leadership style (*n* = 6), coaching leadership style (*n* = 6), affiliate leadership style (*n* = 6), democratic leadership style (*n* = 6), commanding leadership style (*n* = 6), and isolating leadership style (*n* = 6) variables were collapsed into sum variables (*n* = 6) and divided by the number of variables. Next, sum variables were rounded to full figures. The mean SD and minimum and maximum values were calculated for the new sum variables. The internal consistency of the sum variables was assessed with the coefficient Cronbach alpha; values varied from 0.6 to 0.7 [30]. Correlations between variables were analysed using cross-tabulation and chi-squared test. One-way analysis of variance was used to reveal differences in means of sum variables in background variable groups.

## 3. Results and Discussion

### 3.1. Participants' Background Data

Almost all participants were female (97.6%) with an average age of 50.50 years (SD = 7.096). Over half of the nurse managers (61.6%) were specialized nurses, and 15.1% had undertaken higher vocational training. The majority of the respondents (94.0%) had participated in updating education in leadership. The majority of the participants had worked in health care for more than 15 years (93.4%). They had an average of 26.31 years (SD = 7.715) of working experience in health care. The average duration of working experience as a nurse manager was 10.21 years (SD = 7.934). Most of the nurse managers (94%) had participated in updating education. Some nurse managers (13) reported that they led more than one health centre ward. The average number of patients was 34.96 (SD = 14.064), and the average number of employees was 25.58 (SD = 11.777). 

### 3.2. Nurse Managers' Leadership Styles and Their Perceptions of the Importance of the Different Leadership Styles

All six leadership styles—visionary, coaching, affiliate, democratic, commanding, and isolating—were reflected on. The findings of the mean scores for the leadership styles as indicated in Table 2 revealed that visionary leadership style received the highest rating (*m* = 4.51), and isolating leadership style received the lowest rating (*m* = 2.41). The results of the nurse managers' perceptions of the importance of different leadership styles show that almost all respondents in every age group considered four leadership styles—visionary, coaching, affiliate, and democratic—as being very important or important (Table 3). The number of employees was not statistically significantly linked with any of the leadership styles.

Over half of the nurse managers (65.6%) reported that they considered visionary leadership style to be very important. The rest of the respondents reported visionary leadership to be important (33.6%) or rather important (0.8%). Regardless of respondents' education, over 60% reported that visionary leadership style is very important. 73.1% of nurse managers with more than 15 years of working experience considered visionary leadership style to be very important.

55.7% of the respondents reported coaching leadership style to be very important. It was most frequently reported to be very important by nurse managers over the age of 55 (69.7%) and least frequently by those aged 51 to 55 years (41.1%). Of the nurse managers with nurse education, 57.9% reported coaching leadership style to be very important. The importance of coaching leadership style was recognized more often by nurse managers who had participated in updating education in leadership (*P* = 0.000). 63% of the respondents with 10 to 15 years of work experience as a nurse manager considered coaching leadership style to be very important. Less than half of the nurse managers (43.1%) reported affiliate leadership style to be very important, while more than half (55.3%) in all age groups considered it important. Of the nurse managers with nurse education, 52.6% reported affiliate leadership style to be very important, while 35.1% of the nurse managers with M.S. education reported it to be very important. Regardless of respondents' length of work experience as nurse manager, 42.4% considered affiliate leadership style to be very important, while 56.0% considered it important.

A democratic leadership style was reported to be very important by half of the respondents (51.7%). It was most frequently (60%) reported to be very important by nurse managers under the age of 46 years and least frequently by those aged from 51 to 55 years (41.8%). 56.6% of the nurse managers with nurse education reported democratic leadership style to be very important, while 43.2% of nurse managers with M.S. education reported it to be very important. Regardless of respondents' length of work experience as nurse manager, 50.8% reported democratic leadership style to be very important and 48.8% to be important.

Over half of the nurse managers reported commanding leadership style to be rather important (61.1%), while 15.4% considered it rather unimportant. Regardless of respondents' education, almost 65% reported that commanding leadership style is rather important. 22.2% of the nurse managers with 5 to 9 years of work experience considered commanding leadership style not at all important. Regardless of respondents' age, education, or length of work experience as nurse manager, isolating leadership style was reported to be rather important by half of the respondents (53.9%) and rather unimportant by 43%.

### 3.3. Nurse Managers' Perceptions of Their Skills and Knowledge in Leadership Styles

Few nurse managers (7.2%) reported that they did not have at all sufficient skills and knowledge, while two-thirds of the nurse managers (64%) reported that they did not have quite sufficient skills and knowledge in visionary leadership style. Not quite sufficient skills and knowledge in this leadership style were reported by one in ten of the nurse managers aged from 32 to 45 years (9.1%), nurse managers with nurse education (10.7%), and nurse managers with less than 5 years of work experience as nurse manager (10.1%). Nurse managers' skills and knowledge in visionary leadership style became better with the increasing work experience (*P* = 0.005).

Two-thirds (64.8%) of the nurse managers thought they had rather sufficient skills and one-fourth thought (25.6%) they had sufficient skills and knowledge in coaching leadership style. One in every ten respondents (9.8%) reported that they did not have quite sufficient skills and knowledge in this leadership style, while 11.4% of the respondents with specialized nurse education reported this. It was most frequently reported by nurse managers who had less than 5 to 9 years of work experience as nurse managers (17.5%).

Regardless of age or education, some nurse managers reported that they (11.5%) had not quite sufficient, two-thirds that they (66.7%) had rather sufficient, and almost one-third (28.8%) that they had sufficient skills and knowledge in affiliate leadership style. Older nurse managers had better skills and knowledge in affiliate leadership style than younger nurse managers (*P* = 0.005). Every fifth (20.0%) of the respondents with 5 to 9 years of work experience as nurse manager reported that they did not have quite sufficient skills and knowledge in this style. Nurse managers with long-term work experience as managers had better skills and knowledge in affiliate leadership style than managers with shorter work experience (*P* = 0.003). Managers of big units with a large number of employees had less skills and knowledge in affiliate leadership style than managers of small units (*P* = 0.003).

Almost all of the nurse managers evaluated that they had rather sufficient (54.3%) or sufficient (42.4%) knowledge and skills in democratic leadership style. Nurse managers' updating education in leadership increased their skills and knowledge in democratic leadership style (*P* = 0.005). Having knowledge and skills in commanding leadership style was reported to be rather sufficient by 61.7% of the nurse managers and sufficient by 30%. One tenth (10.0%) of the nurse managers with specialized nurse education reported that they did not have quite sufficient skills and knowledge in this style. Nurse managers with shorter work experience as managers had less skills and knowledge in commanding leadership style than nurse managers with longer work experience (*P* = 0.025).

Over two-thirds (68.5%) of the nurse managers reported that they had sufficient skills in isolating leadership style. Nurse managers' participation in updating education increased their skills and knowledge in isolating leadership style (*P* = 0.003). A summary of the nurse managers' perceptions of their skills and knowledge in leadership styles, and the importance of these styles without background variables is presented in Table 4.

### 3.4. Discussion

Based on data from 246 nurse managers working in health centre wards, this study set out to explore nurse managers' leadership styles and their perceptions of the importance of the different leadership styles and their skills and knowledge in leadership styles. Today, nurse managers have to use leadership styles that are appropriate for the constantly changing and complex health care system [31]. The findings of this study suggest that nurse managers most often used visionary, coaching, affiliate, and democratic leadership styles. Commanding and isolating leadership styles were reported to be less frequently used. A previous study found affiliate and coaching leadership styles to be most common [32]. According to the results, regardless of respondents' length of work experience, over 70% considered visionary, coaching, and affiliate leadership styles to be important. It is remarkable that at the same time, approximately 25% of respondents with less than ten years of work experience as nurse managers reported that they had sufficient skills and knowledge in these leadership styles. 

Considered as a challenge for nursing science and management, evidence-based practice could be seen as an ongoing desirable vision. A key question is how appropriate skills nurse managers have in utilising nursing research findings and translating them into practice [18]. According to earlier research findings, nurse managers wish for more time to articulate their vision [33], while nurses emphasize the importance of making the vision understandable by providing information about current issues [28]. These perceptions probably show a contradiction between the vision of the organization and the skills and resources available for its implementation. 

Nurse managers' skills and knowledge in visionary leadership style improved with the increasing work experience. This is likely due to the fact that when nurse managers have worked longer in the organizations they have become acquainted with the strategy and vision of the organization and may have participated in creating the vision. It is important to bear in mind that especially new nurse managers need education in order to be better able to lead others toward the vision.

There is a generational shift going on among nurses, and young nurses have thoughts of giving up nursing. Their experience of job satisfaction and opportunities for development is poor [6]. Older nurses have their own expectations of work. In a Swedish study, informants described that leadership works when it is built on relationships that contribute to a well-functioning work unit, promoting a positive atmosphere [34]. Trust and job satisfaction have strong links with greater commitment and intent to stay on at work [35]. If nurse managers want to advance collaboration among generations, a positive and understanding attitude is needed. The most important thing is that nurses are committed to their patients, and nurse managers are committed to supporting these nurses, despite generational differences [36]. However, it is important to remember that creating harmony does not eliminate the fact that employees sometimes feel resentment and have different opinions [32]. 

Continuing education of the employees is one of the features of infrastructure the magnet hospital nurse managers emphasized because nurses were not able to keep up-to-date with their profession and newest treatment and care modalities [37]. However, even though attitudes towards a coaching leadership style were positive—almost all respondents considered it to be important or very important—one in ten respondents estimated their skills and knowledge to be insufficient. Health care organizations are undergoing changes, which puts pressure on nurse managers and employees to modify and develop their work, even though it provides opportunities for doing so. At the same time, nursing science and practice are fighting to maintain their position. Therefore, nurse managers with a coaching leadership style need strength and assertiveness in times of change to be able to lead employees in an environment where they are noticed as irreplaceable partners within the organization [17]. 

Nurse managers in Finland usually have nurse education, specialized nurse education, and/or academic education (M.S.). Nurse managers need updating education to develop their own professional abilities. Moreover, they need knowledge of nursing science and practice so as to be able to manage the work unit as a whole. Nurse managers should know how to argue decisions in order to get employees to commit to their work and changes. On the other hand, nurse managers need these skills also when they discuss evidence-based nursing in connection with efficiency (providing nursing care with the appropriate level of staff) and effectiveness (achieving desired outcomes) in nursing [18]. When the organization drafts a new strategy and vision for the future, including nursing practice, the nurse manager is the key person to examine nursing and its resources. 

A democratic, participative leadership style allows nurses to become involved in decisions regarding patient care delivery and cooperation with other personnel groups [37]. This probably increases nurses' job satisfaction and commitment to work. On the contrary, it has been noted that some nurse managers do not stand out as leaders, but as team members. This means that the nurse manager's own tasks could be of secondary importance [28]. However, it is important to remember that there are situations in which nurse managers have to make difficult decisions. Decision-making is facilitated by sufficient knowledge, work experience, and support from colleagues and supervisors. 

In the future, well-motivated, professionally developing nurses are needed in health centre wards in Finland. At the same time, nurse managers will need skills and knowledge to lead their work units in a visionary manner. Work units, employees and situations differ, and it could be said that there is no one and only correct leadership style; the same result can be achieved in many ways. The behaviour of emotionally intelligent leaders stimulates the creativity of their employees [38]. Nurse manager's ability to reflect on their own behaviour makes it easier to regulate and estimate their leadership style with different employees in different situations. It is important to arrange enough updating education to support nurse managers in their leadership work.

### 3.5. Limitations

The questionnaire used in this study was based on the leadership styles presented by Goleman et al. [20] and earlier research of Vesterinen et al. [27, 28]. Goleman et al. [20] described primal leadership, which requires the leader to bring emotional intelligence (EI) to bear on his/her leadership. The complex phenomenon of emotional intelligence in nursing leadership is under the consideration of criticism. It is essential to have profound knowledge of EI and its scientific critique when integrating the concept into nursing research [39]. On the other hand, the content of the questionnaire was evaluated by an expert panel, which consisted of nurse managers working in different types of hospital wards. On the basis of this evaluation, the indicator used in this study can be considered valid in terms of the concepts used for studying nurse managers' leadership styles in health centre hospital wards. The value of Cronbach's alpha used to study the internal consistency between the six sum variables was between 0.6 and 0.7, which could be considered as sufficient [40]. The study has some limitations. The results achieved may not necessarily give a true picture of the nurse managers' skills and knowledge in the use of different leadership styles, as nurse managers' skills and knowledge were only estimated by the nurse managers themselves, not their employees or supervisors. In this study, the sample consisted of Finnish nurse managers; therefore, the results reflect only their perceptions of leadership styles. 

## 4. Conclusions 

Health care organizations are undergoing continuing changes, and vision should be present at all times. Employees need new skills and knowledge to be able to manage their work. At the same time, they hope that their individual needs are taken into account by the nurse managers. Nurse managers' abilities to reflect, understand, and, if necessary, change their leadership style influence a work unit's success and employees' job satisfaction. Nurse managers, especially new nurse managers, need more theoretic, evidence-based education to cope with these expectations and to develop their professional abilities. 

Health care organizations should draw up visions of nursing leadership in the future. Together with universities, they should start planning nurse manager education programmes that focus on strategic issues, leadership, job satisfaction, challenging situations in leadership, change management, work unit management (e.g., economy, efficiency, and resources), and how nurse managers consider their own wellbeing. It is important that nurse managers have peer groups and mentors for helping them to develop as managers. It is health care organizations' responsibility to set up a clear vision and goals and make the successful nurse leadership possible as part of multiprofessional cooperation.

## Figures and Tables

**Table 1:** The respondents' (*n* = 252) background data.

Background data	*n*	%
Gender (*n* = 252)		
Female	246	97.6
Male	6	2.4
Age (*n* = 248)		
32–45	56	22.6
46–50	69	27.8
51–55	56	22.6
56–65	67	27.0
Education (*n* = 245)		
Specialized nurse	151	61.6
Nurse	57	23.3
MSc	37	15.1
Work experience in health care (*n* = 249)		
<21	60	24.1
21–25	53	21.3
26–30	63	25.3
>30	73	29.3
Work experience as nurse manager (*n* = 252)		
<5	71	28.2
5–9	65	25.8
10–14	47	18.7
>14	69	27.3
Updating education in leadership (*n* = 252)		
Yes	237	94.0
No	15	6.0
Reading professional journals (*n* = 252)		
Yes	216	85.7
No	36	14.3
Reading scientific journals (*n* = 252)		
Yes	113	44.8
No	139	55.2
Searching information from internet (*n* = 252)		
Yes	173	68.7
No	79	31.3
Discussing with colleagues (*n* = 252)		
Yes	220	87.3
No	32	12.7
Consulting experts (*n* = 252)		
Yes	91	36.1
No	161	63.9
Number of employees (*n* = 247)		
<20	69	28.2
20–24	66	26.9
25–29	46	18.8
>29	64	26.1

**Table 2:** Nurse managers' leadership styles in health centre wards.

Leadership style	Mean	SD
Visionary	4.51	0.37
Coaching	4.45	0.38
Affiliate	4.33	0.39
Democratic	4.39	0.35
Commanding	2.97	0.55
Isolating	2.41	0.51

**Table 3:** Nurse managers' perceptions of the importance of different leadership styles.

Background data	Very important (%)	Important (%)	Rather important (%)	Rather little important (%)	Not at all important (%)
Visionary leadership style					

Age					
32–45	72.7	25.5	1.8	0.0	0.0
46–50	63.8	34.8	1.4	0.0	0.0
51–55	55.6	44.4	0.0	0.0	0.0
56–65	69.8	30.2	0.0	0.0	0.0

Coaching leadership style					

Age					
32–45	48.2	51.8	0.0	0.0	0.0
46–50	60.3	36.8	2.9	0.0	0.0
51–55	41.1	57.1	1.8	0.0	0.0
56–65	69.7	30.3	0.0	0.0	0.0

Affiliate leadership style					

Age					
32–45	39.3	60.7	0.0	0.0	0.0
46–50	44.9	52.2	2.9	0.0	0.0
51–55	35.7	62.5	1.8	0.0	0.0
56–65	50.8	47.7	1.5	0.0	0.0

Democratic leadership style					

Age					
32–45	60.0	40.0	0.0	0.0	0.0
46–50	55.2	43.3	1.5	0.0	0.0
51–55	41.8	58.2	0.0	0.0	0.0
56–65	49.2	50.8	0.0	0.0	0.0

Commanding leadership style					

Age					
32–45	0.0	30.4	60.7	8.9	0.0
46–50	1.5	7.6	72.7	18.2	0.0
51–55	0.0	17.9	57.1	25.0	0.0
56–65	0.0	22.2	68.3	9.5	0.0

Isolating leadership style					

Age					
32–45	0.0	0.0	49.0	51.0	0.0
46–50	0.0	3.0	38.8	58.2	0.0
51–55	0.0	1.9	38.9	59.3	0.0
56–65	0.0	5.2	46.6	46.6	1.7

**Table 4:** Nurse managers' perceptions of their skills and knowledge on leadership styles.

	How adequate are my knowledge and skills?			
	Not at all sufficient	Not quite sufficient	Rather Sufficient	sufficient
Visionary leadership style	7.2%	64.0%	28.8%	0.0%
Coaching leadership style	0.0%	9.6%	64.8%	25.6%
Affiliate leadership style	0.0%	11.5%	66.7%	28.8%
Democratic leadership style	0.0%	3.2%	54.2%	42.6%
Commanding leadership style	0.0%	8.8%	61.2%	30.0%
Isolating leadership style	0.4%	1.2%	29.9%	68.5%

## References

[1] Huston C (2008). Preparing nurse leaders for 2020. *Journal of Nursing Management*.

[2] Carney M (2009). Leadership in nursing: current and future perspectives and challenges. *Journal of Nursing Management*.

[3] Spence Laschinger HK, Wilk P, Cho J, Greco P (2009). Empowerment, engagement and perceived effectiveness in nursing work environments: does experience matter?. *Journal of Nursing Management*.

[4] Statistics Finland Population projection 2009–2060. http://www.stat.fi/til/vaenn/2009/vaenn_2009_2009-09-30_tie_001_en.html.

[5] National Institute for Health and Welfare Sosiaali-ja terveydenhuollon henkilöstö 2007. http://www.stakes.fi/tilastot/tilastotiedotteet/2010/Tr07_10.pdf.

[6] Flinkman M, Laine M, Leino-Kilpi H, Hasselhorn HM, Salanterä S (2008). Explaining young registered Finnish nurses’ intention to leave the profession: a questionnaire survey. *International Journal of Nursing Studies*.

[7] Espinoza DC, Lopez-Saldana A, Stonestreet JS (2009). The pivotal role of the nurse manager in healthy workplaces: implications for training and development. *Critical Care Nursing Quarterly*.

[8] Cowden T, Cummings G, Profetto-Mcgrath J (2011). Leadership practices and staff nurses’ intent to stay: a systematic review. *Journal of Nursing Management*.

[9] Farag AA, Tullai-Mcguinness S, Anthony MK (2009). Nurses’ perception of their manager’s leadership style and unit climate: are there generational differences?. *Journal of Nursing Management*.

[10] Tomey AM (2009). Nursing leadership and management effects work environments. *Journal of Nursing Management*.

[11] Casida J, Parker J (2011). Staff nurse perceptions of nurse manager leadership styles and outcomes. *Journal of Nursing Management*.

[12] Huber DL, Maas M, McCloskey J, Scherb CA, Goode CJ, Watson C (2000). Evaluating nursing administration instruments. *Journal of Nursing Administration*.

[13] Suhonen M, Paasivaara L (2011). Shared human capital in project management: a systematic review of the literature. *Project Management Journal*.

[14] Teperi J, Porter ME, Vuorenkoski L, Baron JF (2009). The Finnish health care system: a value-based perspective. *Sitra Reports*.

[15] Ministry of Social affairs and Health 2010 Terveydenhuoltolaki. http://www.stm.fi/vireilla/lainsaadantohankkeet/sosiaali_ja_terveydenhuolto/terveydenhuoltolaki.

[16] National Institute for Health and Welfare Terveyskeskusten perusterveydenhuollon vuodeosastohoito 2008. http://www.stakes.fi/tilastot/tilastotiedotteet/2010/Tr01_10.pdf.

[17] Watters S (2009). Shared leadership: taking flight. *Journal of Nursing Administration*.

[18] Newhouse RP (2007). Creating infrastructure supportive of evidence-based nursing practice: leadership strategies. *Worldviews on Evidence-Based Nursing*.

[19] Weberg D (2010). Transformational leadership and staff retention: an evidence review with implications for healthcare systems. *Nursing Administration Quarterly*.

[20] Goleman D, Boyatzis R, McKee A (2002). *Primal Leadership Realizing the Power of Emotional Intelligence*.

[21] Akerjordet K, Severinsson E (2008). Emotionally intelligent nurse leadership: a literature review study. *Journal of Nursing Management*.

[22] Feather R (2009). Emotional intelligence in relation to nursing leadership: does it matter?. *Journal of Nursing Management*.

[23] Young-Ritchie C, Spence Laschinger HK, Wong C (2009). The effects of emotionally intelligent leadership behaviour on emergency staff nurses’ workplace empowerment and organizational commitment. *Nursing Leadership*.

[24] McCallin A, Bamford A (2007). Interdisciplinary teamwork: is the influence of emotional intelligence fully appreciated?. *Journal of Nursing Management*.

[25] Lucas V, Spence Laschinger HK, Wong CA (2008). The impact of emotional intelligent leadership on staff nurse empowerment: the moderating effect of span of control. *Journal of Nursing Management*.

[26] Vesterinen S, Isola A, Paasivaara L (2007). Hoitotyön johtajien johtamistyylitutkimus vuosina 1994-2006. *Premissi*.

[27] Vesterinen S, Isola A, Paasivaara L (2009). Leadership styles of Finnish nurse managers and factors influencing it. *Journal of Nursing Management*.

[28] Vesterinen S, Suhonen M, Isola A, Paasivaara L (2012). Nurse managers’ leadership styles in Finland. *Nursing Research and Practice*.

[29] Burns N, Grove SK (2001). *The Practise of Nursing Research*.

[30] Munro B (2001). *Statistical Methods For the Health Care Research*.

[31] Casida J, Pinto-Zipp G (2008). Leadership-organizational culture relationship in nursing units of acute care hospitals. *Nursing Economics*.

[32] Kenmore P (2008). Exploring leadership styles. *Nursing Management*.

[33] Graham IW, Jack E (2008). Promoting leadership: the development of a nurse executive team in an acute hospital trust. *Journal of Nursing Management*.

[34] Rosengren K, Athlin E, Segesten K (2007). Presence and availability: staff conceptions of nursing leadership on an intensive care unit. *Journal of Nursing Management*.

[35] Way C, Gregory D, Davis J (2007). The impact of organizational culture on clinical managers’ organizational commitment and turnover intentions. *Journal of Nursing Administration*.

[36] Mensik JS (2007). A view on generational differences from a generation X leader. *Journal of Nursing Administration*.

[37] Upenieks VV (2003). What constitutes effective leadership?: perceptions of magnet and nonmagnet nurse leaders. *Journal of Nursing Administration*.

[38] Rego A, Sousa F, Pina e Cunha M, Correia A, Saur-Amaral I (2007). Leader self-reported emotional intelligence and perceived employee creativity: an exploratory study. *Creativity and Management*.

[39] Akerjordet K, Severinsson E (2010). The state of the science of emotional intelligence related to nursing leadership: an integrative review. *Journal of Nursing Management*.

[40] Polit D, Hungler B (1997). *Essentials of Nursing Research, Methods, Appraisal and Utilization*.

